# Political Partisanship and Antiscience Attitudes in Online Discussions About COVID-19: Twitter Content Analysis

**DOI:** 10.2196/26692

**Published:** 2021-06-14

**Authors:** Ashwin Rao, Fred Morstatter, Minda Hu, Emily Chen, Keith Burghardt, Emilio Ferrara, Kristina Lerman

**Affiliations:** 1 Information Sciences Institute University of Southern California Marina del Rey, CA United States

**Keywords:** COVID-19, Twitter, infodemiology, infodemic, infoveillance, multidimensional polarization, social media, social network

## Abstract

**Background:**

The novel coronavirus pandemic continues to ravage communities across the United States. Opinion surveys identified the importance of political ideology in shaping perceptions of the pandemic and compliance with preventive measures.

**Objective:**

The aim of this study was to measure political partisanship and antiscience attitudes in the discussions about the pandemic on social media, as well as their geographic and temporal distributions.

**Methods:**

We analyzed a large set of tweets from Twitter related to the pandemic, collected between January and May 2020, and developed methods to classify the ideological alignment of users along the moderacy (hardline vs moderate), political (liberal vs conservative), and science (antiscience vs proscience) dimensions.

**Results:**

We found a significant correlation in polarized views along the science and political dimensions. Moreover, politically moderate users were more aligned with proscience views, while hardline users were more aligned with antiscience views. Contrary to expectations, we did not find that polarization grew over time; instead, we saw increasing activity by moderate proscience users. We also show that antiscience conservatives in the United States tended to tweet from the southern and northwestern states, while antiscience moderates tended to tweet from the western states. The proportion of antiscience conservatives was found to correlate with COVID-19 cases.

**Conclusions:**

Our findings shed light on the multidimensional nature of polarization and the feasibility of tracking polarized opinions about the pandemic across time and space through social media data.

## Introduction

Effective response to a health crisis requires society to forge a consensus on many levels: scientists and doctors have to learn about the disease and quickly and accurately communicate their research findings to others, public health professionals and policy experts have to translate the research into policies and regulations for the public to follow, and the public has to follow guidelines to reduce infection spread. However, the fast-moving COVID-19 pandemic has exposed our critical vulnerabilities at all these levels. Instead of orderly consensus-building, we have seen disagreement and controversy that exacerbated the toll of the disease. Research papers are rushed through the review process, with results sometimes being disputed or retracted [1], policy makers giving conflicting advice [2], and scientists and many in the public disagreeing on many issues, from the benefits of therapeutics [3] to the need for lockdowns and face-covering [4]. The conflicting viewpoints create conditions for polarization to color perceptions of the pandemic [5-8] and attitudes toward mitigation measures.

Surveys have identified a partisan gulf in the attitudes about COVID-19 and the costs and benefits of mitigation strategies, with the public’s opinion polarized into sharply contrasting positions. According to a Pew Research Center report [9], political partisanship significantly affects perceptions of public health measures and might explain regional differences in the pandemic’s toll in the United States [10]. Polarization has colored the messages of US political leaders about the pandemic [7] as well as discussions of ordinary social media users [8]. Coupled with a distrust of science and institutions, polarization can have a real human cost if it leads the public to minimize the benefits of face coverings or reject the COVID-19 vaccine when it becomes available. Dr Anthony Fauci, the nation’s top infectious diseases expert, attributed many of the disease’s 500,000 deaths (and counting) to political divisions in the country [11]. This further affirms the need to investigate the presence, and unravel the ill effects, of polarization in scientific and political discourse.

Current research measures polarization as divergence of opinions along the political dimension and its effect on other opinions, for example, discussion of scientific topics [12]. However, opinions on controversial issues are often correlated [13]; for example, those who support transgender rights also believe in marriage equality, and those who oppose lockdowns also resist universal face-covering. Inspired by this idea, we capture some of the complexity of polarization by projecting opinions in a multidimensional space, with different axes corresponding to different semantic dimensions. Once we identify the dimensions of polarization and define how to measure them, we can study the dynamics of polarized opinions, their interactions, and regional differences.

Our work analyzed tweets posted on Twitter related to the COVID-19 pandemic collected between January 21 and May 1, 2020 [5]. We studied polarization along three dimensions: political (liberal vs conservative), science (proscience vs antiscience), and moderacy (hardline vs moderate). User polarization along the science axis identifies whether users align with scientific and factual sources of information or whether they are characterized by mistrust of science and preference for pseudoscientific and conspiracy sources. A user’s political ideology is defined in a 2D space. Working in tandem with the political axis, the moderacy dimension recognizes the intensity of partisanship from hardline to moderate. For the hardliners identified along the moderacy dimension, we leveraged the political axis to identify their partisanship as liberal or conservative.

Cinelli et al [14] and Weld et al [15] showed that sharing of URLs annotated by Media Bias/Fact Check is a reliable proxy of one’s political polarity. Inspired by the findings and conclusions made in these works, we used media sources that have been classified by nonpartisan sites along these dimensions to define the poles of each dimension of polarization. These media sources include both mainstream news and a large variety of other sources, such as government agencies, nongovernmental organizations, crowdsourced content, and alternative medicine news and health sites. Users were given a score reflecting how often they shared information from each set of polarized sources. These users served as training data to train machine learning algorithms to classify remaining users along the multiple dimensions of polarization based on the content of their posts. Inferring the polarization of users discussing COVID-19 allowed us to study the relationships between polarized ideologies and their temporal and geographic distributions. We showed that political and science dimensions were highly correlated and that politically hardline users were more likely to be antiscience, while politically moderate users were more often proscience. We also identified regions of the United States and time points where the different ideological subgroups were comparably more active and we identified their topics of conversation. We found that areas of heightened antiscience activity corresponded to US states with large COVID-19 outbreaks. Our work, therefore, provides insights into potential reasons for geographic heterogeneity of outbreak intensity.

The contributions of this work are as follows:

- We described a framework to infer the multidimensional polarization of social media users, allowing us to track political partisanship and attitudes toward science at scale.
- We showed that political and science dimensions were highly correlated, with hardline right and antiscience attitudes closely aligned.
- We studied the geographical distribution of polarized opinions and found that regional differences can correlate with the pandemic’s toll.

As the amount of COVID-19 information explodes, we need the ability to proactively identify emerging areas of polarization and controversy. Early identification could lead to more effective interventions to reduce polarization and also improve the efficacy of disease mitigation strategies. Vaccine hesitancy was shown in past research to be associated with antiscience attitudes [16]; therefore, our approach may help identify regions of the country that will be more resistant to COVID-19 vaccination. This may better prepare public health workers to target their messages.

## Methods

Here, we describe the data and methods we used for measuring polarization and also inferred it from text and online interactions.

### Data Set

In this study, we used a public data set of COVID-19 tweets from Twitter [5]. This data set comprises 115 million tweets from users across the globe, collected over a period of 101 days from January 21 to May 1, 2020. These tweets contain at least one keyword from a predetermined set of COVID-19–related keywords (eg, coronavirus, pandemic, and Wuhan).

Fewer than 1% of the tweets in the original corpus have geographic coordinates associated with them. We specifically focused on tweets from users located in the United States, at state-level granularity, based on geolocated tweets and fuzzy matching of user profile text [8]. Specifically, we used a fuzzy text matching algorithm to detect state names and abbreviations, as well as names of populous cities. The user profile text extracted from the *description* attribute of the *user* object was passed on to the *loc_to_state* function of the georeferencing code [17] to extract the user’s location at the state level. A manual review of this approach found it to be effective in identifying the user’s home state. This methodology provided location information for 65% of users in the data set. The georeferenced data set consisted of 27 million tweets posted by 2.4 million users over the entire time period.

### Measuring Polarization Using Domain Scores

We characterized individual attitudes along three dimensions of polarization. The *political* dimension, the standard dimension for characterizing partisanship, captured the difference between *left* (*liberal*) and *right* (*conservative*) stances for users with strong hardline political opinions. The *science* dimension captured an individual’s acceptance of evidence-based *proscience* views or the propensity to hold *antiscience* views. People believing and promoting conspiracies, especially health-related and pseudoscientific conspiracies, were often grouped in the antiscience camp. Finally, the *moderacy* dimension described the intensity of partisanship, from *moderate* or nonpartisan opinions to politically *hardline* opinions.

We inferred polarized attitudes of users from the content of their posts. While previous work [18] inferred polarization from user hashtags, we instead relied on user-tweeted URLs. The key idea that motivated our approach is that online social networks tend to be ideologically similar, with users more closely linked (eg, through follower relationships) to others who share their beliefs [19,20]. While we did not have follow links in our data, we used URLs as evidence [21] of a homophilic link. We extended this approach beyond political ideology [22] to label other dimensions of polarization. Specifically, we used a curated list of information sources, whose partisan leanings were classified by neutral websites, to infer the polarization of Twitter users at scale. We used lists compiled by Media Bias/Fact Check, AllSides, and NewsGuard, which tracks coronavirus misinformation (see data folder at GitHub [23]). Table 1 lists exemplar domains, hereinafter referred to as pay-level domains (PLDs), in each category. PLDs listed under *conspiracy* and *questionable sources* were mapped to our antiscience category. For the *moderacy* axis, we considered the union of left and right PLDs as a proxy for the *hardline* category, while the union of least-biased, left-moderate, and right-moderate PLDs formed the proxy *moderate* category.

We quantified a user’s position along the dimensions of polarization by tracking the number of links to curated PLDs the user shared. Specifically, we extracted PLDs that were shared by users in the data set and filtered for relevant PLDs that were present in our curated lists (Table 1). This gave us a set of 136,000 users who shared *science* PLDs, 169,000 users who shared *political* PLDs, and 234,000 users who shared PLDs along the *moderacy* dimension. There was a wide distribution in the number of tweets, and therefore PLDs, shared between users (Figure S1 in Multimedia Appendix 1), with some users tweeting many PLDs and many users tweeting one or none. We, therefore, filtered out users who shared fewer than *three* relevant PLDs in each dimension (ie, fewer than three in the science dimension, fewer than three in the partisan dimension, and fewer than three in the moderacy dimension), which resulted in 18,700 users. For each user, we computed a *domain score δ* along each of the three dimensions, as the average of mapped domain values of a dimension:

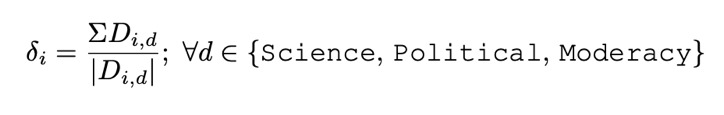



where *δ_i_* is the domain score of *user_i_* and *D_i,d_* represents the set of PLDs shared by *user_i_* relevant to dimension *d*.

**Table 1:** Curated information and news pay-level domains (PLDs) with their polarization.

Dimension and polarization dimension		PLDs, n	Examples of PLDs
**Science^a^**			
	Proscience (+1)	150+	cdc.gov, who.int, thelancet.com, mayoclinic.org, nature.com, and newscientist.com
	Antiscience (−1)	450+	911truth.org, althealth-works.com, naturalcures.com, shoebat.com, and prison-planet.com
**Political^b^**			
	Liberal (−1)	300+	democracynow.org, huffingtonpost.com, newyorker.com, occupy.com, and rawstory.com
	Conservative (+1)	250+	nationalreview.com, newsmax.com, oann.com, theepochtimes.com, and bluelivesmatter.blue
**Moderacy^c^**			
	Moderate (+1)	400+	ballotpedia.org, c-span.org, hbr.org, wikipedia.org, weforum.org, snopes.com, and reuters.com
	Hardline (−1)	500+	gopusa.com, cnn.com, democracynow.org, huffingtonpost.com, oann.com, and theepochtimes.com

^a^Proscience PLDs are mapped to +1 along the science axis, while antiscience PLDs are mapped to −1.

^b^Along the political axis, liberal PLDs are mapped to −1, while conservative PLDs are mapped to +1.

^c^Along the moderacy axis, hardline PLDs are mapped to −1, while moderate PLDs are mapped to +1.

**Figure 1:**
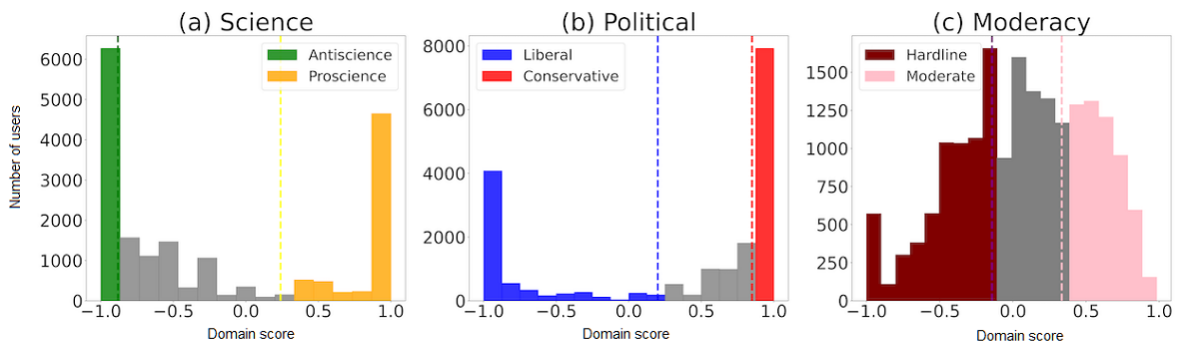
The distribution of domain scores along science, political, and moderacy dimensions. (a) The vertical lines at 0.42 and −1 mark the top and bottom 30% cutoffs of distribution along the science dimension, which are binned as proscience (+1) and antiscience (−1), respectively. (b) The vertical lines at 1 and −0.33 mark the top and bottom 30% cutoffs of distribution along the political dimension, which are binned as conservative (+1) and liberal (−1), respectively. (c) The vertical lines at 0.38 and −0.18 mark the top and bottom 30% cutoffs of distribution along the moderacy dimension, which are binned as moderate (+1) and hardline (−1), respectively.

Figure 1 shows the distribution of domain scores for users who shared links to information sources across all dimensions. The distributions were peaked at their extreme values, showing more users sharing information from antiscience than proscience PLDs and more conservative than liberal PLDs. In Figure S2 in Multimedia Appendix 1, we show that these extremes were robust to how we filtered users and were, therefore, not a product of, for example, sharing a single link.

For network-level analysis, we then built a web scraper that mapped PLDs to their respective Twitter handles. The scraper initiated a simple Google query of the form “*Domain Name Twitter Handle.*” This tool relied on the search engine to rank results based on relevance and picked out the title of the first result containing the substring “|*Twitter.*” This substring was of the form “*Account Name* (@*handle*) | *Twitter,*” which was parsed to retrieve the domain’s corresponding handle. We manually verified the mapped PLDs. The mapped dimension-wise PLDs are available on our GitHub repository under the data folder.

Recall that along each of the three dimensions, we mapped the dimension’s constituent domain names to their respective Twitter handles. The mapped Twitter handles formed our seed sets for semisupervised learning at the network level. Each dimension’s seed set comprised key-value pairs of Twitter handles and their corresponding orientation along the dimension. Table 2 illustrates the number of seeds along each polarization axis.

To investigate the presence of bias stemming from an uneven distribution of PLDs along each ideological dimension’s polarized ends, we sampled an equal number of PLDs along each of the dimension’s two polarities. More specifically, we performed random downsampling of the majority ideological polarity along each dimension. Upon ensuring that each dimension’s polarized ends were now represented by an equal number of PLDs, we calculated domain scores for each user along the ideological dimensions (Figure S3 in Multimedia Appendix 1). Leveraging these domain scores, we then rebuilt prediction models. We found that the performance of this modified procedure did not differ significantly from our results (see Table S1 in Multimedia Appendix 1 for more details). This robustness check demonstrated the versatility of our approach to differences in the sampling of PLDs along each dimension.

**Table 2:** Description of the retweet network.

Dimension and polarization		Seeds^a^, n (%)
**Science (n=158)**		
	Proscience	81 (51.3)
	Antiscience	77 (48.7)
**Political (n=195)**		
	Liberal	96 (49.2)
	Conservative	99 (50.8)
**Moderacy (n=558)**		
	Hardline	195 (34.9)
	Moderate	363 (65.1)

^a^Number of seed handles along each polarization axis for initial node assignment in the label propagation algorithm.

### Inferring Polarization

#### Overview

Using domain scores, we were able to quantify the polarization of just a small fraction (18,700/2,400,000, 0.78%) of users who generated PLDs in the data set. In this section, we describe how we leveraged this data to infer the polarization of the remaining users in our data set. In the Results section, we compare the performance of these inference methods. Two methods, label propagation algorithm (LPA) and latent Dirichlet allocation (LDA), act as baselines against our state-of-the-art text embedding method. Our study focused on investigating content generated by users over the entire period rather than at the noisier tweet level. Investigating a user’s content, tweet by tweet, may or may not provide sufficient information to gauge their ideological polarity, whereas analyzing all tweets generated by a user over time would facilitate this.

We classified users according to the binned domain scores along each dimension. We found that classification worked better than regression in this data set. We binned domain scores by thresholding the distribution into two classes along each dimension, as shown in Figure 1. Using other threshold values to bin the domain score distribution into two classes did not qualitatively change results (Multimedia Appendix 1). Additionally, we released a GitHub repository [23] for readers to reproduce this work upon careful rehydration of tweet data, instructions for which have also been provided in the repository.

#### Label Propagation Algorithm

LPA was used in the past to label user ideology based on the ideology of accounts the user retweets (eg, see Badawy et al [22]). The idea behind label propagation is that people prefer to connect to, and retweet content posted by, others who share their opinions [24,25]. This gives us an opportunity to leverage topological information from the retweet network to infer users’ propensity to orient themselves along ideological dimensions.

The geocoded Twitter data set provides fields named *screen_name* and *rt_user,* which allowed us to identify the user being retweeted and the user retweeting, respectively. To this end, we built a network from 9.8 million retweet interactions between 1.9 million users sourced from the data set. In the retweet network, an edge runs from *A* to *B* if user *A* retweets user *B*. Descriptive statistics of the retweet network are shown in Table 3. We then used the semisupervised greedy learning algorithm (ie, the LPA) to identify clusters in the retweet network.

LPA, as proposed by Raghavan et al [26], is a widely used near-linear time node classification algorithm. This greedy learning method started off with a small set of labeled nodes also known as seeds, with the remaining nodes assigned labels at random. The number of seeds for each polarization dimension is shown in Table 2. The algorithm then iteratively updated the labels of nonseed nodes to the majority label of their neighbors, with ties broken at random, until converging to an equilibrium where the labels no longer changed. However, owing to stochasticity of tie-breaking, a certain amount of randomness crept into the results produced by this algorithm. As the result, LPA tended to generate slightly differing classifications of user polarization for the same network each time it was run. To address the stochasticity, we ran the LPA in 5-fold cross-validation and averaged the results.

**Table 3:** Statistics of the network.

Statistic	Value, n
Nodes	1,857,028
Maximum in-degree	39,149
Maximum out-degree	1450
Retweets	9,788,251
Unique retweets	7,745,533
Size of the strongly connected component	1,818,657

#### Latent Dirichlet Allocation

We used LDA [27] to identify topics, or groups of hashtags, and represented users as vectors in this topic space. We considered the set of *all* hashtags in the COVID-19 data set generated by a user over time as a document representing that user—after ignoring hashtags used by fewer than 10 users or more than 75% of the users—leaving 25,200 hashtags. The choice of 75% was arbitrary, but a hashtag that appeared at a lower threshold (eg, within roughly 50% of the users) could be highly prevalent in one domain and not another. We used a more lenient threshold to avoid this issue. We also used 20 topics, as that gave a higher coherence score. Given the enormity of the geocoded Twitter data set we leveraged in this study, conducting LDA experiments to validate these thresholds proved to be computationally prohibitive and it was unlikely that tuning would have achieved significantly better results than the one seen in this study.

We used the document-topic affinity matrix generated by LDA to represent users. An *affinity vector* was composed of 20 likelihood scores corresponding to 20 topics, adding up to 1, with each score indicating the probability of the corresponding topic being a suitable representation for the set of hashtags generated by the user. Using these affinity vectors, we generated feature vector matrices for each of the three dimensions of interest. In doing so, we were able to represent over 900,000 users who used some hashtag in their tweets with a dense vector of length 20.

#### Text Embedding Using fastText

Previous methods—see Conover et al [28]—classified a user’s political polarization based on the text of their tweets by generating term frequency–inverse document frequency–weighted unigram vectors for each user. However, the advent of more powerful text-embedding techniques [29-31] allowed us to generate sentence-embedding vectors to better represent content.

We grouped the tweets generated by each of the 2.4 million users from January to May 2020. More specifically, we collected all COVID-19–related tweets generated by a user in this time period and concatenated them to form a text document for each user. After preprocessing the 2.4 million documents to lowercase and removing hashtags, URLs, mentions, handles, and stop words, we used the fastText sentence-embedding model pretrained on Twitter data to generate tweet embeddings for each user. Preprocessing of tweets was performed by leveraging the regular expression (*re*) package in Python, version 3.7 (Python Software Foundation); the Natural Language Toolkit; and the *Gensim* natural language processing library. The *Sent2vec* Python package [32] provided us with a Python interface to quickly leverage the pretrained model and generate 700-dimension feature vectors representing each user’s discourse.

## Results

### Overview

First, we visualized the domain scores of the 18,700 users, showing the relationship between the science, moderacy, and political dimensions. Then we compared the performance of algorithms for classifying users along the three dimensions of polarization, using domain scores as ground truth data. We used the inferred scores to study the dynamics and spatial distribution of polarized opinions of users engaged in online discussions about COVID-19.

### Visualizing Polarization

**Figure 2:**
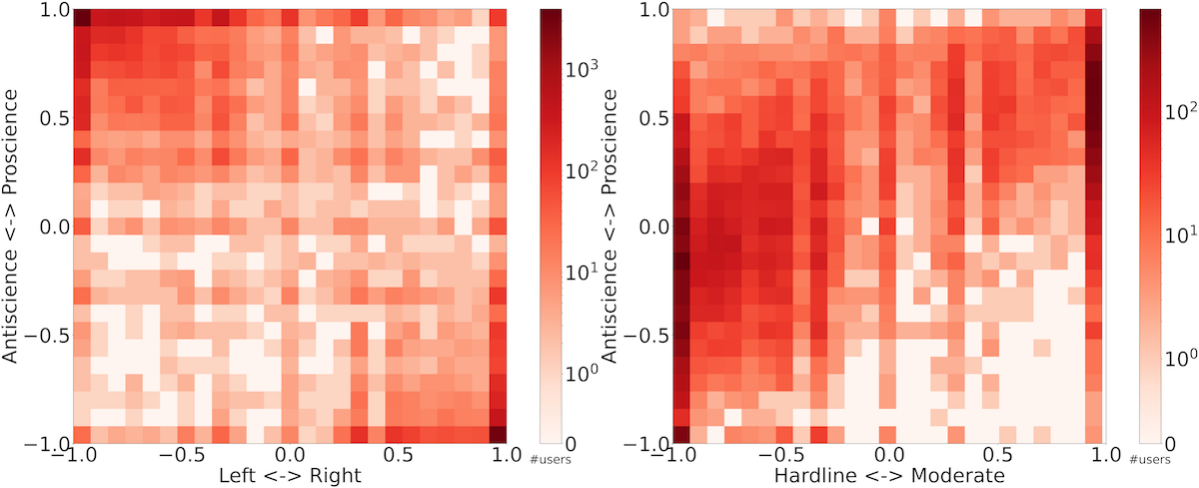
Polarization of COVID-19 tweets. On the left is the heat map of polarization (domain scores) along the science-partisanship dimensions. On the right is the heat map of polarization (domain scores) along the science-moderacy dimensions. Each bin within the heat map represents the number of users with domain scores falling within that bin.

Figure 2 shows the relationship between dimensions of polarization, leveraging domain scores of 18,700 users who shared information from curated PLDs. The heat map shows the density of users with specific domain scores. Large numbers of users are aligned with proscience-left extreme (top-left corner) or antiscience-right extreme (bottom-right corner), with lower densities along the diagonal between these extremes (Figure 2, left-hand side). This illustrates the strong correlation between political partisanship and scientific polarization, thereby highlighting the influence of pernicious political divisions on evidence-based discourse during the pandemic, with conservatives being more likely to share antiscience information than proscience sources. The heat map on the right-hand side in Figure 2 highlights the interplay between the science and moderacy axes. The white region in the bottom-right corner shows there are few antiscience users who are politically moderate, thus demonstrating an asymmetry in these ideologies. The shading also highlights a higher density of proscience users identifying as politically moderate. These results are robust to how data are filtered, as shown in Figure S4 in Multimedia Appendix 1.

### Classifying Polarization

To run the LPA, we started from a set of labeled seeds: Twitter handles corresponding to PLDs categorized along the dimensions of interest (Tables 1 and 2). We reserved some of the seeds along each dimension for testing LPA predictions and reported accuracy of 5-fold cross-validation.

For content-based approaches, we used binned domain scores of 18,700 users as ground truth data to train logistic regression models to classify user polarization along the three dimensions. We represented each user as a vector of features generated by different content-based approaches: topic vectors for LDA and sentence embeddings for the fastText approach. We reserved a subset of users for testing performance.

Table 4 compares the performance of polarization classification methods. LPA worked well when it tried to identify user alignment along the political and science dimensions. However, it failed to capture the subtler distinctions along the moderacy axis. Training was further hampered by the low number of retweet interactions with moderate PLDs in comparison to hardline ones. Of the 1.8 million retweet interactions, only 250,000 involved some moderate seed nodes, whereas over 1 million interactions involved some hardline seed nodes. Moreover, poor classification performance with LPA revealed an important pattern: that moderates surrounded themselves with diverse opinions and, thus, a clear distinction could not be made by observing who they retweeted.

LDA modeling on hashtags allowed us to generate reduced-dimension, dense feature vectors for over 900,000 users who used hashtags in their tweets. This representation allowed us to design better learning models that significantly outperformed the LPA model.

A logistic regression model trained on fastText outperformed all other models described in this study. Coupled with fastText’s ability to better handle out-of-vocabulary terms, the model’s access to finer levels of detail at the tweet-text level, culminated in it being able to better predict dimensions of polarization. Given the model’s superior performance across all three dimensions, we leveraged its predictions in subsequent analyses. We classified users along the three polarization dimensions. However, since the definition of the hardline extreme of the moderacy dimension overlapped with the political dimension, we needed to report only six ideological groups, rather than all eight combinations.

**Table 4:** Performance of polarization classification.^a^

Method and dimension		Data set size, n	Accuracy, %	Precision, %	Recall, %	F1 score, %
**Label propagation algorithm**						
	Science	158	92*.*6	*100* ^b^	80	88*.*9
	Political	195	92*.*3	86*.*9	*100*	93*.*0
	Moderacy	1205	20*.*1	72	1*.*4	2*.*74
**Latent Dirichlet allocation**						
	Science	9983	92*.*2	91*.*6	92*.*4	91*.*9
	Political	11,020	93*.*5	95*.*1	93*.*3	94*.*2
	Moderacy	9565	86*.*4	85*.*6	85*.*0	85*.*4
**fastText**						
	Science	11,202	*93.8*	93*.*9	*93.7*	*93.8*
	Political	12,425	*95.1*	*96.5*	94*.*6	*95.5*
	Moderacy	11,197	*90.2*	*90.1*	*90.5*	*90.2*

^a^Results compare classification performance of the label propagation algorithm and content-based methods, including topic modeling (latent Dirichlet allocation) and full-text embedding (fastText). Results are averages of 5-fold cross-validation. Data set sizes are the number of users in model validation data sets and are composed of users with strong polarization scores (top or bottom 30% as defined previously) in the filtered 18,700-user data set.

^b^Values in italics indicate the best-performing models.

## Discussion

### Dynamics of Polarization

Research shows that opinions of Twitter users about controversial topics do not change over time [33]. To investigate whether user alignments along the three polarization dimensions changed over time, we grouped tweets by time into seven biweekly intervals: January 21 to 31, 2020; February 1 to 15, 2020; February 16 to 29, 2020; March 1 to 16, 2020; March 17 to 31, 2020; April 1 to 15, 2020; and April 16 to May 1, 2020. There were 3000 users who tweeted consistently in all seven biweekly intervals. For each of the *N* users, we computed cumulative domain scores along science, political, and moderacy dimensions for all time intervals *t* and computed the average absolute change 
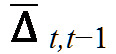
 in domain score from biweekly period *t*−1 along each dimension given by the following:

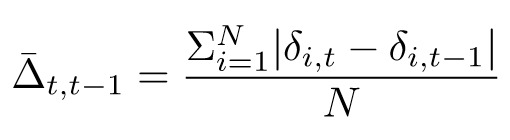



where *δ_i,t_* represents the domain score for a user *i* in biweekly period *t*. The small values of 
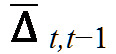
 in Table 5 confirm that user alignments do not change significantly over time.

**Table 5:** Average absolute change in domain score along consecutive biweekly intervals.

Dimension	Average absolute change ( 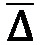 ) per biweekly interval numbers					
	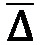 2*,*1	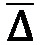 3*,*2	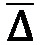 4*,*3	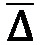 5*,*4	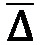 6*,*5	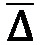 7*,*6
Political	0*.*09	0*.*05	0*.*03	0*.*02	0*.*03	0*.*02
Science	0*.*13	0*.*07	0*.*04	0*.*02	0*.*02	0*.*02
Moderacy	0*.*21	0*.*11	0*.*07	0*.*04	0*.*04	0*.*03

**Figure 3:**
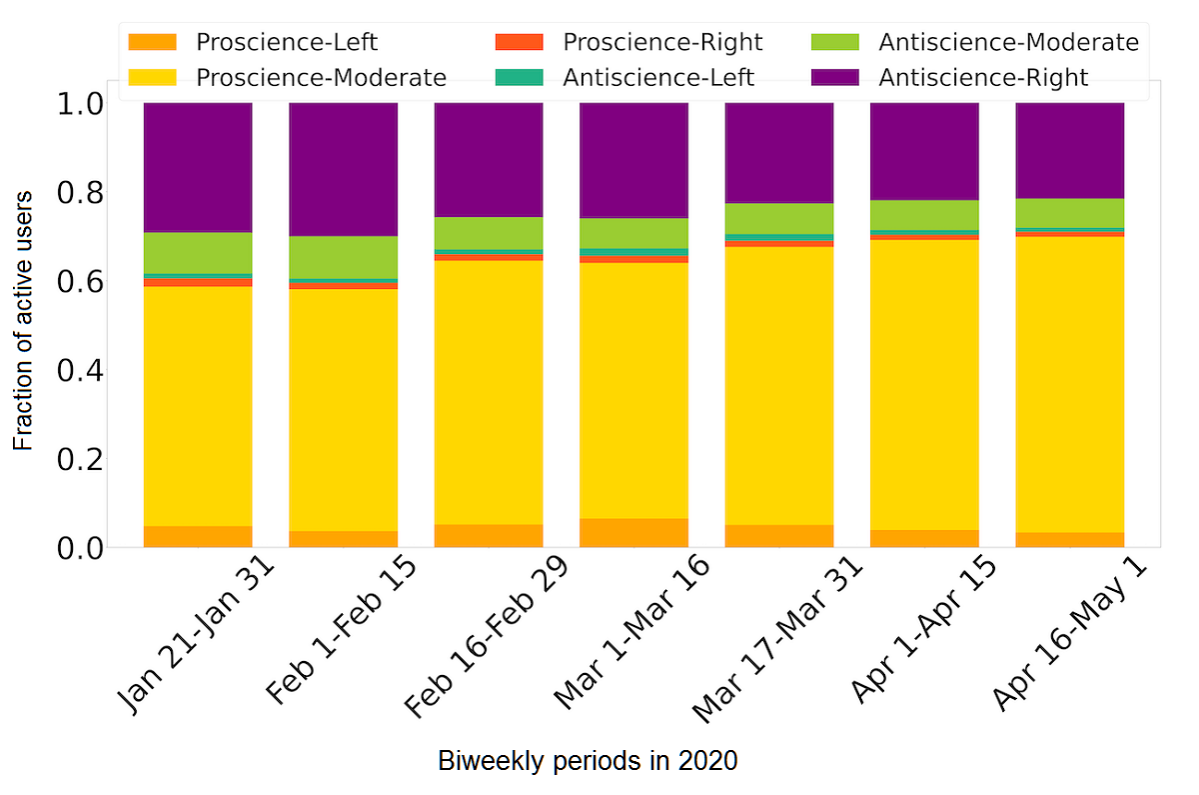
Fraction of active users per ideological group in biweekly periods. For completeness, this plot shows all users in the data set and not the filtered 18,700 users.

Although each individual’s alignments did not change, the number of users within each ideological group did change over time. User alignments did not change; therefore, we leveraged polarization classification results to show biweekly fractions of active users per ideological category. Figure 3 shows the composition of active users in all categories. As time progressed, we could clearly see the growth in the proscience-moderate category accompanied by a corresponding decline in antiscience-right users. This was consistently found over a variety of data filters, as seen in Figure S5 in Multimedia Appendix 1.

### Topics of Polarization

**Figure 4:**
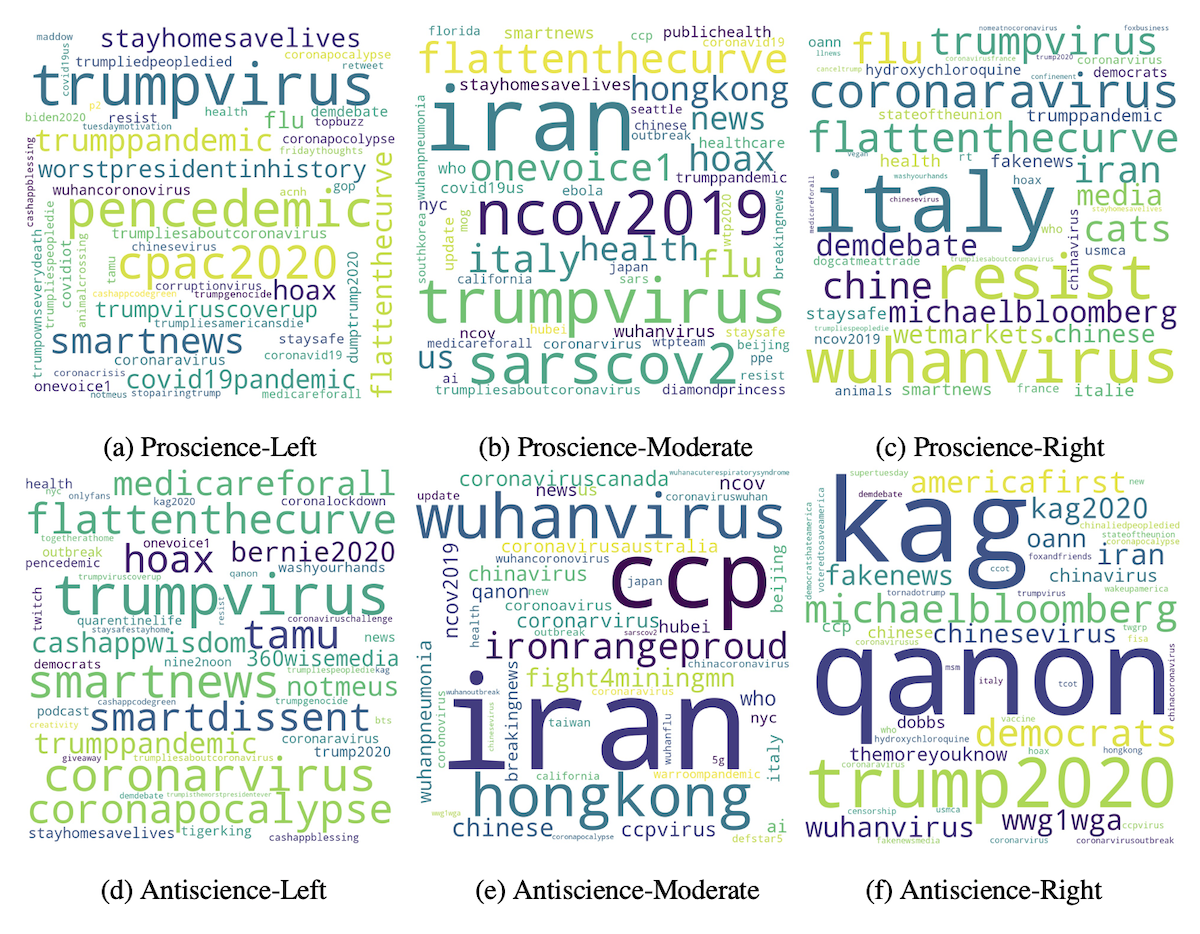
Topics of discussion within the six ideological groups. The top row (from left to right) illustrates topics for proscience-left, proscience-moderate, and proscience-right groups. The bottom row (from left to right) illustrates topics for antiscience-left, antiscience-moderate, and antiscience-right groups.

To better understand what each of the six groups tweeted about, we collected the 50 most frequent hashtags used by each group, after removing hashtags common to all six groups. Figure 4 shows the word clouds of the most common hashtags within each group, sized by the frequency of their occurrence. Most striking was the use of topics related to conspiracy theories, such as *#qanon* and *#wwg1wga* by the antiscience-right group, along with politically charged references to the *#ccpvirus* and *#chinavirus*. This group also used hashtags related to former US President Donald Trump’s re-election campaign, showing the hyper-partisan nature of COVID-19 discussions. Another partisan issue appeared to be *#hydroxychloroquine*, a drug promoted by Donald Trump. It showed up in both proscience-right and antiscience-right groups but was not discussed by other groups. Overall, these intuitive results highlight the overall accuracy of our polarization inference model.

The polarized nature of the discussions could be seen in the users of the hashtags *#trumppandemic* and *#trumpvirus* by the left and proscience groups. However, in contrast to antiscience groups, proscience groups talked about COVID-19 mitigation strategies, using hashtags such as *#stayhomesavelives*, *#staysafe*, and *#flattenthecurve*.

### Geography of Polarization

**Figure 5:**
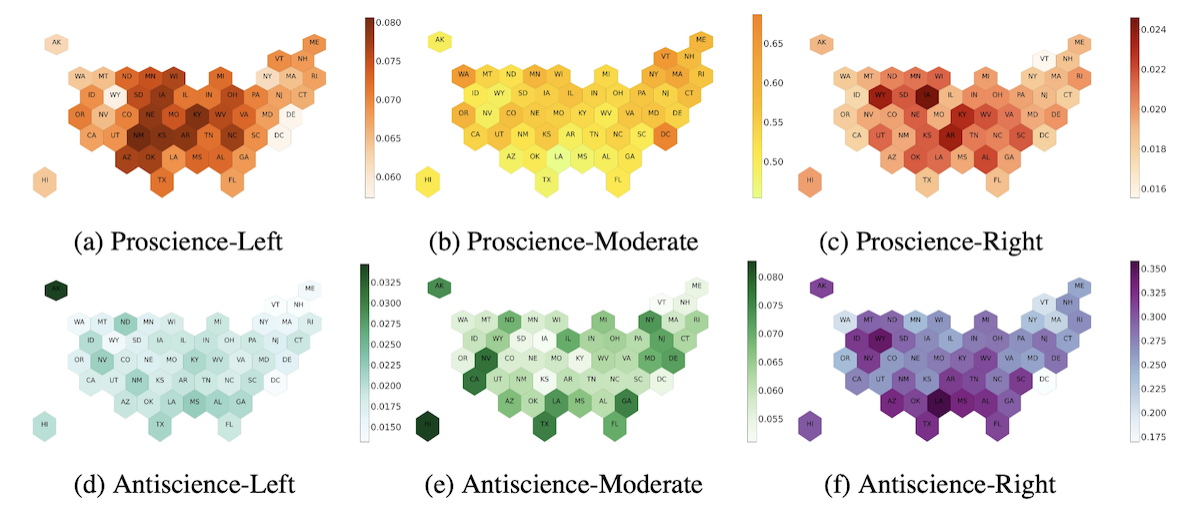
Fraction of US states' Twitter users per ideological category. Plots (a) to (c) (top row, left to right) show the fraction of states' Twitter users who were classified as proscience-left, proscience-moderate, and proscience-right, respectively. Plots (d) to (f) (bottom row, left to right) show the fraction of states’ Twitter users who were classified as antiscience-left, antiscience-moderate, and antiscience-right, respectively. The vertical bars next to the maps indicate the fraction of Twitter users in the state belonging to the ideological group. Two-letter abbreviations are used for each state.

Responses to the coronavirus pandemic in the United States have varied greatly by state. While the governors of New York, California, Ohio, and Washington reacted early by ordering lockdowns, the governors of Florida and Mississippi have downplayed the gravity of the situation for a longer time. To explore the geographical variation in ideological alignments, we grouped users by the state from which they tweeted and computed the fraction of their respective state’s Twitter users belonging to an ideological group. We then generated geo-plots, shown in Figure 5, to highlight the ideological composition of each state.

We saw a higher composition of proscience-moderates, as seen in Figure 5 (b), in Washington, Oregon, DC, and Vermont. As expected, these states had a lower fraction of antiscience users, as can be seen from Figure 5 (d), (e), and (f). Governors of these states were quick to enforce lockdowns and spread pandemic awareness among the general public.

Over the course of the pandemic, we have seen the strong opposition to masking mandates and closing down of businesses in California, Nevada, Hawaii, Georgia, and Texas. These antiscience sentiments are reflected in Figure 5 (e), which shows that these states had a comparatively higher proportion of their Twitter users in the antiscience-moderate ideology group.

Southern states—South Carolina, Mississippi, Louisiana, Texas, and Arizona—and northwestern states—Wyoming, North Dakota, South Dakota, and Montana—have experienced COVID-19 surges, with southern states becoming overwhelmed during the summer of 2020 and northwestern states becoming overwhelmed in the fall of 2020 (Figure S6 in Multimedia Appendix 1 shows the cumulative COVID-19 cases per state). The political and religious leaders in these states have also consistently downplayed the pandemic and resisted mitigation strategies. Our results are consistent with this, showing that these states also had more conservative Twitter users who mistrust science, as manifested by sharing information from antiscience sources. The antiscience attitudes in these states may also spell trouble for vaccination plans. The statistically significant positive correlation (Figure S7 in Multimedia Appendix 1) between state-wise cumulative COVID-19 case counts and antiscience-right users, as well as the negative correlation between the former and proscience-moderate users, affirms the significance of scientific beliefs in mitigating the spread of virus.

### Limitations and Future Directions

Our novel approach to identify ideological alignments of users on Twitter comes with certain limitations. Akin to other studies involving Twitter data, our study worked under the caveat that the behaviors of the subset of users being considered in our data set may not be representative of population behavior. The use of geolocation techniques and subsequent consideration of users with a geolocation could introduce certain biases, which necessitate further investigation.

Thresholds that were used in our LDA analysis of user hashtags have been set intuitively due to LDA’s prohibitive computation needs when dealing with over 900,000 hashtags. It is unlikely that we would have observed significant improvements in classification results with different thresholds. However, we encourage readers to investigate this further.

Additionally, the seed sets (Table 2) employed for our label propagation experiments may have had room for bias, as not all PLDs collected had a corresponding Twitter account. The cross-section of PLDs that have a Twitter account could be biased by political orientation, age group that the PLD caters to, etc. Investigation of bias stemming from this is a promising prospect for future work. Furthermore, our analyses worked under the assumption that media bias ratings provided by Media Bias/Fact Check accurately exhibited ideological biases of media sources. Leveraging these ratings, we assumed that generating tweets consisting of PLDs was an expression or reflection of one’s ideological polarity. Future studies can build on these assumptions, and interesting avenues can be explored by incorporating other indicators of user polarity.

Verification of agreement or disagreement of user viewpoint and content in PLDs being shared was not in the purview of this study, and we encourage our readers to explore these avenues in future research. Furthermore, although we showed good performance on classifying polarized opinions, additional work is required to infer finer-grained opinions. Namely, by predicting fine-grained polarization among users, we could better infer, for example, network effects, such as whether users prefer to interact with more polarized neighbors, which may adversely impact provaccine mitigation strategies. Moreover, longer-term trends need to be explored in order to better understand how opinions change dynamically. This will better test whether social influence or selective formations of ties are the drivers of echo chambers and polarization. Finally, there is a need to explore polarization across countries to understand how different societies and governments are able to address polarization and how these polarized dimensions relate to one another across the world.

### Conclusions

Our analysis of a large corpus of online discussions about COVID-19 confirms and extends the findings of opinion polls and surveys [9]: opinions about COVID-19 are strongly polarized along partisan lines. Political polarization strongly interacts with attitudes toward science: conservatives are more likely to share antiscience information related to COVID-19, while liberal and more moderate users are more likely to share information from proscience sources. On the positive side, we found that the number of proscience, politically moderate users dwarfed other ideological groups, especially antiscience groups. This is reassuring from the public health point of view, suggesting that a plurality of people are ready to accept scientific evidence and trust scientists to lead the way out of the pandemic. The geographical analysis of polarization identified regions of the country, particularly in the south and the west where antiscience attitudes are more common, that correlate to areas with particularly high COVID-19 cases, as seen in Figure S6 in Multimedia Appendix 1. Messaging strategies should be tailored in these regions to communicate with science skeptics. Overall, we found that analysis of tweets, while less representative than surveys, offers inexpensive, fine-grained, and real-time analysis of polarization and partisanship.

## Appendix

Multimedia Appendix 1 Supplementary materials.

## Abbreviations

DARPA: Defense Advanced Research Projects Agency
LDA: latent Dirichlet allocation
LPA: label propagation algorithm
PLD: pay-level domain
