# Supplementary material for: Political Partisanship and Antiscience Attitudes in Online Discussions About COVID-19: Twitter Content Analysis
Source: J Med Internet Res. 2021 Jun 14;23(6):e26692. doi: 10.2196/26692 (PMC8204937; doi:10.2196/26692)
Supplement: Multimedia Appendix 1 [file jmir_v23i6e26692_app1.docx]

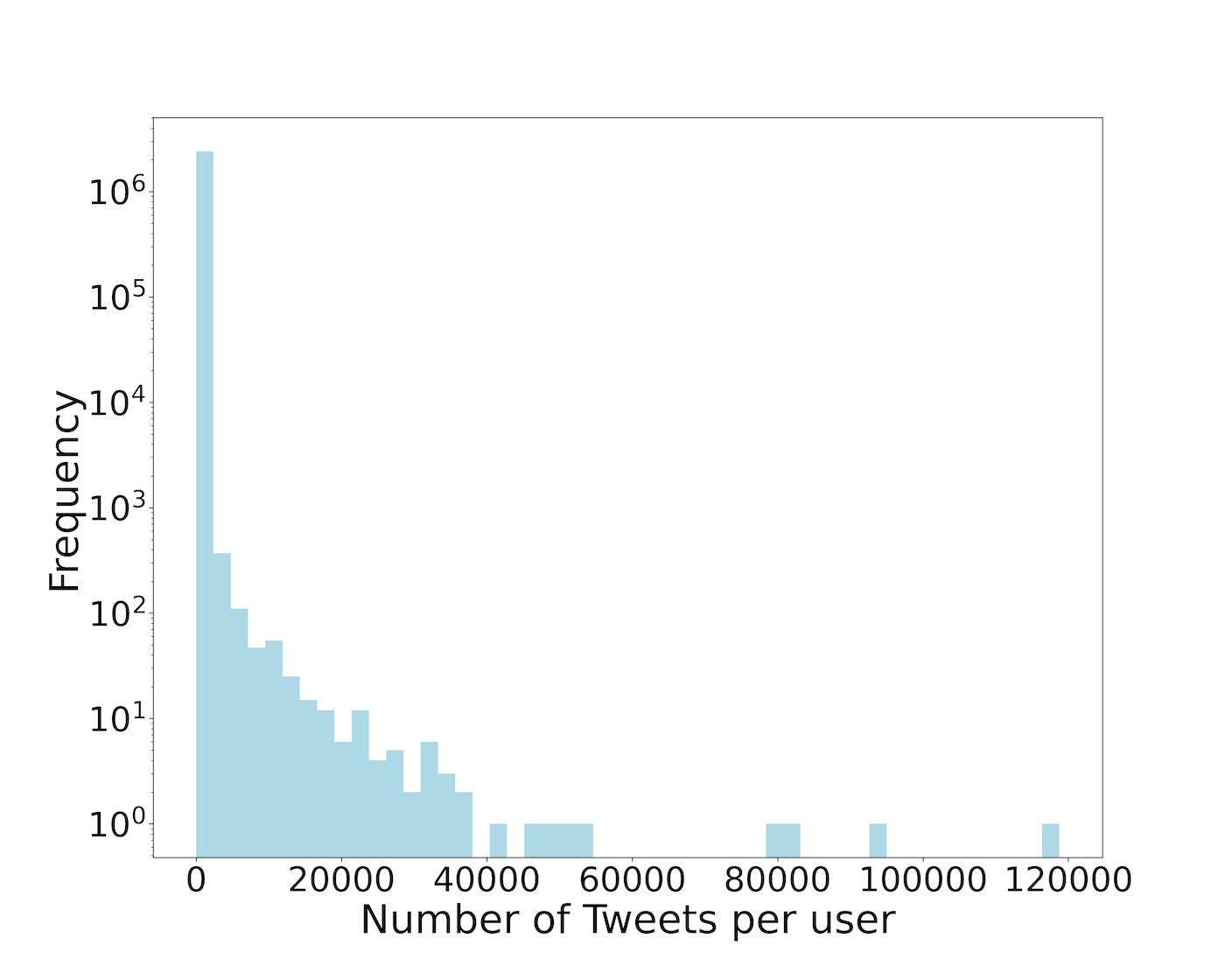


Figure S1: Distribution of user activity, measured as number of tweets per user.


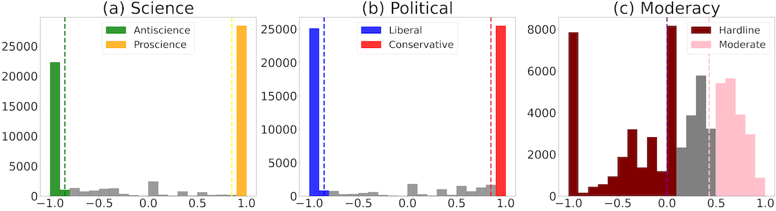


1. Threshold=0


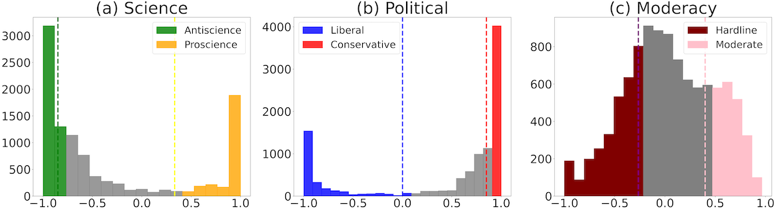


1. Threshold=4


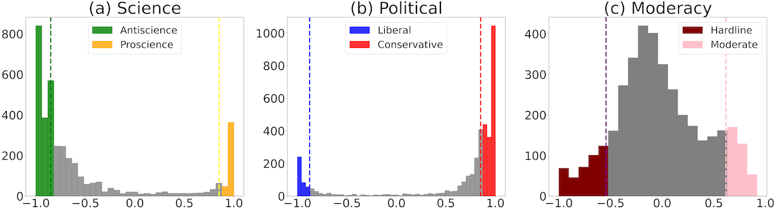


1. Threshold=10

Figure S2: Distribution of user level domain scores across different thresholds on minimum number of PLDs generated per user. Figure(a) shows the user level distribution of domain scores along all three dimensions with threshold of PLDs/user set at 0. Figure(b) shows the distribution for the threshold set at 4. Figure(c) shows the distribution for the threshold set at 10.


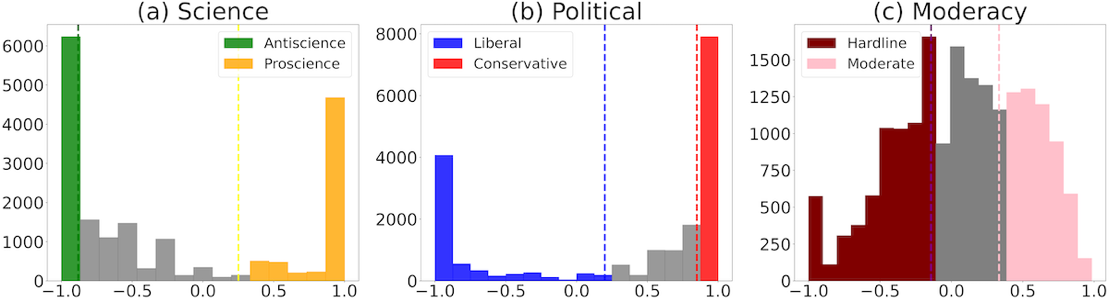


Figure S3: Distribution of user level domain scores calculated by considering an equal number of Pay-Level domains from both ends of each of the three dimensions. Comparing the above figure to Figure 1, we can conclude that the resulting tendencies visible in Figure 1 are a representative reflection of Twitter user’s attitudes.

| **Method** | **Dimension** | **Dataset Size** | **Accuracy** | **Precision** | **Recall** | **F1-Score** |
| --- | --- | --- | --- | --- | --- | --- |
|  | Science | 9963 | 92*.*8% | 92*.*3% | 92*.*9% | 92*.*6% |
| LDA | Political | 10994 | 94*.*2% | 95*.*4% | 94*.*7% | 95*.*0% |
|  | Moderacy | 9511 | 86*.*1% | 85*.*8% | 85*.*3% | 85*.*5% |
|  | Science | 11173 | **94.9%** | **95.4%** | **94*.*2%** | **94.8%** |
| **fastText** | Political | 12392 | **95.0%** | **95*.*5%** | **95*.*5%** | **95.5%** |
|  | Moderacy | 11166 | **90.8%** | **91*.*6%** | **90*.*2%** | **90.9%** |
|  |  |  |  |  |  |  |

Table S1: Performance of Polarization Classification upon considering equal number of Pay-Level domains from both ends of each of the three dimensions in ground truth domain score computation. As LPA did not leverage domain scores it was excluded from this robustness check. We observe that results do not significantly differ from what was observed in Table 4.


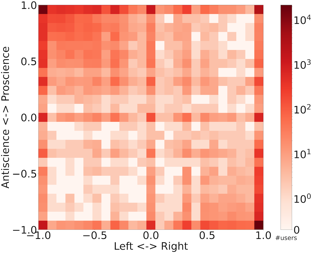

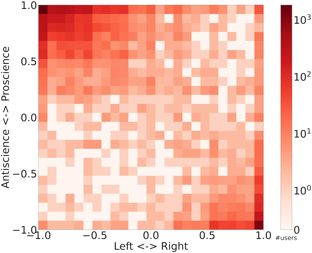

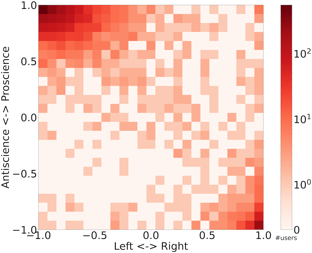


(a) Threshold=0 (b) Threshold=4 (c) Threshold=10


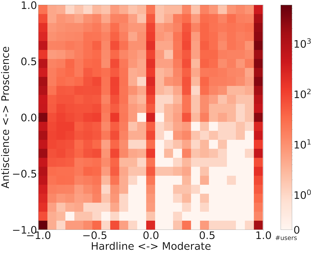

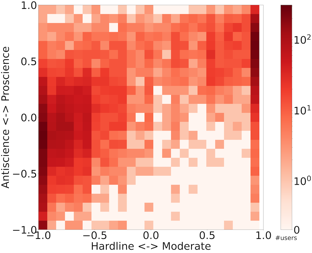

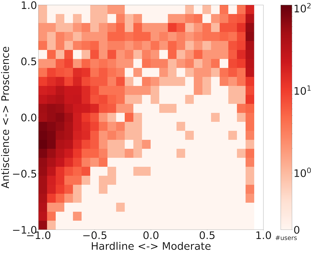


(a) Threshold=0 (b) Threshold=4 (c) Threshold=10

Figure S4: Multidimensional Polarization Heatmaps - Figures (a–c) show the domain score distribution of users along the Science and Political dimensions for number of PLDs thresholds 0,4 and 10. (d–f) show the domain score distribution of users along the Science and Moderacy dimensions for number of PLDs thresholds 0,4 and 10.


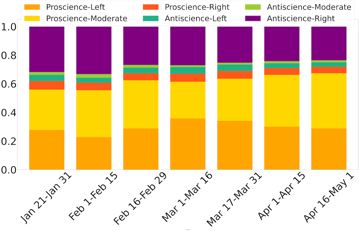

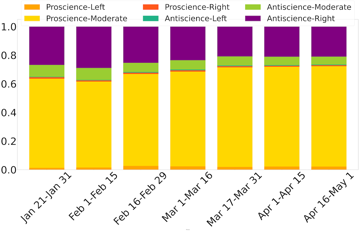

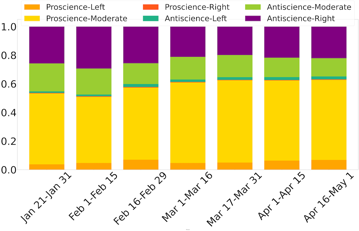


(a) Threshold=0 (b) Threshold=4 (c) Threshold=10

Figure S5: Polarization over time for various number of domain thresholds. (a) Threshold = 0 (all data), (b) threshold= 4 PLDs per dimension, and (c) threshold= 10 PLDs per dimension. We see a higher percentage of Pro-Science Left users in (a) where we set the minimum number of PLDs above zero. The higher percentage of Pro-Science-Left users can be attributed to the model learning from users who share only one domain. As we increase the threshold, we see a more stable presence of Pro-Science Moderate users.


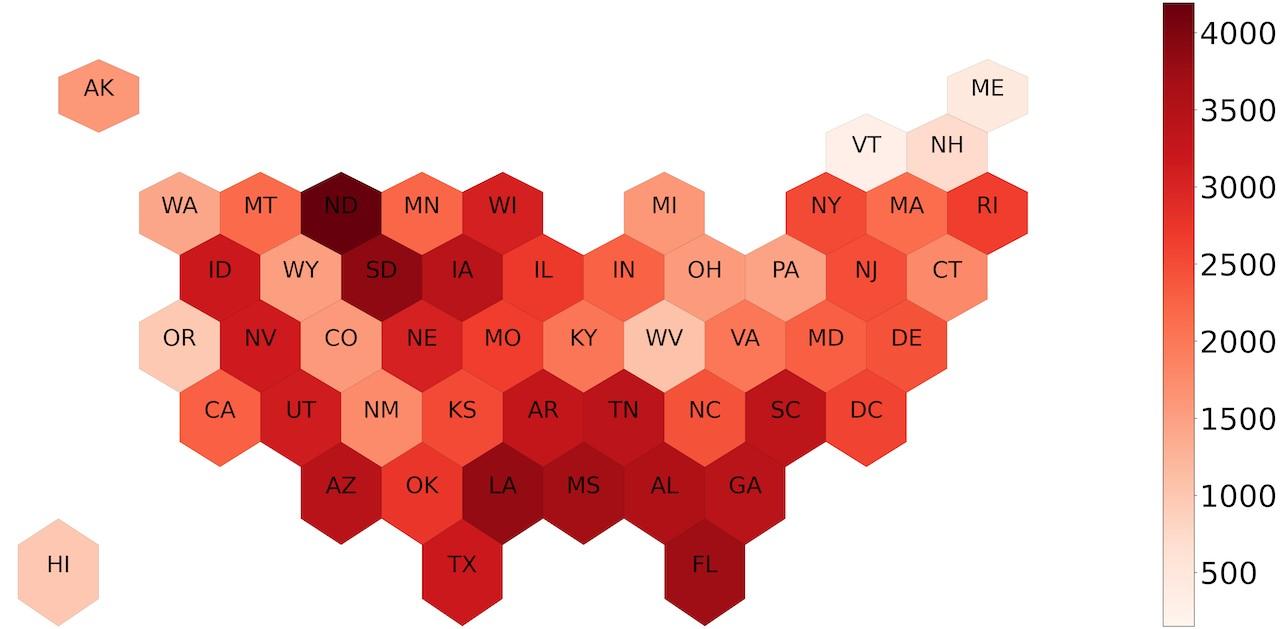


Figure S6: Cumulative COVID-19 cases per 100,000 residents for US states as of October 17, 2020. The map shows the unequal impact of the pandemic on different regions of the US.


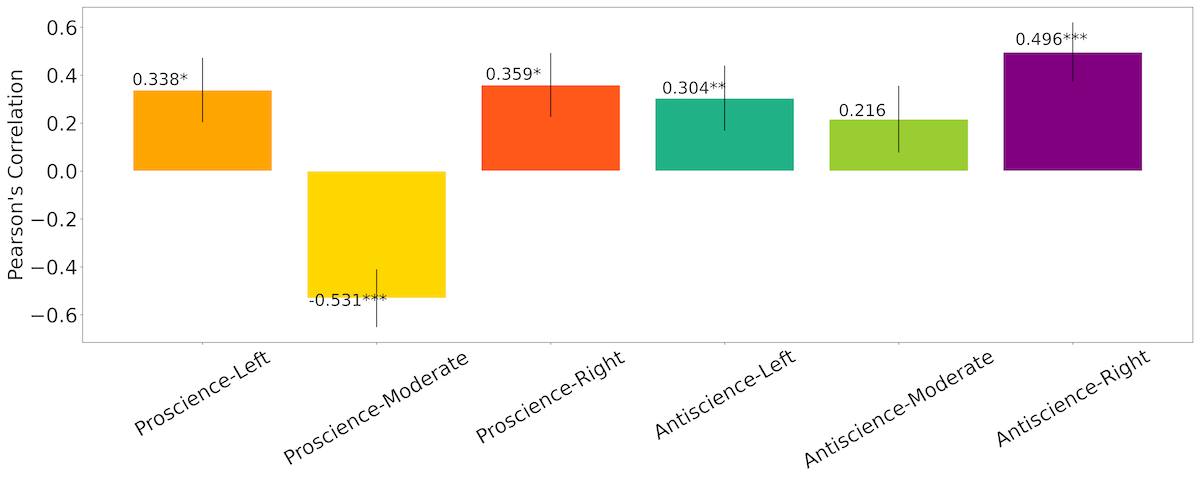


Figure S7: Correlation of state wise cumulative COVID-19 case counts and fraction of Twitter users by ideological category. *** denotes *P*-value < 0*.*001, ** denotes *P*-value = 0*.*002 and * denotes *P*-value = 0*.*03.
